# *Xenopus laevis il11ra.L* is an experimentally proven interleukin-11 receptor component that is required for tadpole tail regeneration

**DOI:** 10.1038/s41598-022-05954-w

**Published:** 2022-02-03

**Authors:** Shunya Suzuki, Kayo Sasaki, Taro Fukazawa, Takeo Kubo

**Affiliations:** Department of Biological Sciences, Graduate School of Science, The University of Tokyo, Tokyo, Japan

**Keywords:** Cell biology, Developmental biology

## Abstract

*Xenopus laevis* tadpoles possess high regenerative ability and can regenerate functional tails after amputation. An early event in regeneration is the induction of undifferentiated cells that form the regenerated tail. We previously reported that *interleukin-11* (*il11*) is upregulated immediately after tail amputation to induce undifferentiated cells of different cell lineages, indicating a key role of *il11* in initiating tail regeneration. As Il11 is a secretory factor, Il11 receptor-expressing cells are thought to mediate its function. *X. laevis* has a gene annotated as *interleukin 11 receptor subunit alpha* on chromosome 1L (*il11ra.L*), a putative subunit of the Il11 receptor complex, but its function has not been investigated. Here, we show that nuclear localization of phosphorylated Stat3 induced by Il11 is abolished in *il11ra.L* knocked-out culture cells, strongly suggesting that *il11ra.L* encodes an Il11 receptor component. Moreover, knockdown of *il11ra.L* impaired tadpole tail regeneration, suggesting its indispensable role in tail regeneration. We also provide a model showing that Il11 functions via *il11ra.L*-expressing cells in a non-cell autonomous manner. These results highlight the importance of *il11ra.L*-expressing cells in tail regeneration.

## Introduction

Organ regenerative ability varies among animal species. Among vertebrates, fish and amphibians possess high regenerative ability, whereas mammals have restricted regenerative ability^1–3^. *Xenopus laevis* tadpoles have high regenerative ability, and can regenerate functional whole tails within a week of amputation^3–5^. In *X. laevis* tadpole tail regeneration, the spinal cord and notochord regenerate from the same tissue type in the stump, and myofibers arise from satellite cells and not from the pre-existing myofibers^6,7^. A recent single-cell resolution analysis of tail regeneration revealed no evidence for the emergence of multipotent progenitors or intermediate cell states suggesting transdifferentiation^8^. These findings suggest that lineage-restricted tissue stem cells or precursors are major contributors to tail regeneration in *X. laevis* tadpoles^6–9^. After tail amputation, these precursors are activated to generate a mass of undifferentiated proliferating cells at the wound stump to form the regeneration bud, and then differentiate to the tissues from which they are derived^6,7^. Induction of undifferentiated proliferating cells is an early and indispensable event for organ regeneration^10^. We previously reported a factor involved in this process; *interleukin-11* (*il11*), which is highly expressed in the regeneration bud, but not in the intact tail or developmental tail bud^11^, is essential for inducing undifferentiated sensory neurons, notochord, and muscle during tail regeneration^12^. Immediate early expression of *il11* occurs within 2 h post amputation (hpa), and undifferentiated cells of different cell lineages are induced only by *il11*, suggesting that it initiates regeneration^12^.

Il11 is a member of the interleukin-6 (Il6) type cytokine family, whose signaling cascade is well described in mammals^13–19^. Il11 is received by the Il11 receptor complex comprising Il11 receptor subunit alpha (Il11ra) and glycoprotein 130 (Gp130; also known as interleukin-6 signal transducer, Il6st), a common co-receptor for the Il6 type cytokine family^13–16^. Binding of Il11 to the Il11 receptor complex activates Janus kinase (Jak), which is associated with Gp130 and phosphorylates Stat3 (signal transducer and activator of transcription 3), and then phosphorylated Stat3 (P-Stat3) translocates to the nucleus to activate downstream transcription^17–19^.

Il11 activity in tissue regeneration is thought to be elicited by cells receiving the secreted Il11, and thus we hypothesized that Il11 receptor-expressing cells play pivotal roles in regeneration. Although *X. laevis* has a gene annotated as *interleukin-11 receptor subunit alpha* on chromosome 1L (*il11ra.L*), its function as an Il11 receptor has not been investigated. We performed a functional analysis of *il1ra.L* in culture cells and in vivo, and an expression analysis of intact tadpoles and regenerating tails. We offer a possible model of *il11ra.L*-expressing cell function in tadpole tail regeneration.

## Results and discussion

### ***X. laevis*** Il11ra.L functions as an Il11 receptor component in cultured cells

First, we investigated whether *X. laevis* Il11ra.L functions as an Il11 receptor component. Il11 elicits Stat3 phosphorylation and its nuclear translocation in mammals^13–19^. We therefore examined whether P-Stat3 nuclear translocation occurs when *X. laevis* Il11 is applied to *X. laevis* cells expressing *il11ra.L*, and whether P-Stat3 nuclear translocation is abolished by *il11ra.L* knockout (KO).

**Figure 1:**
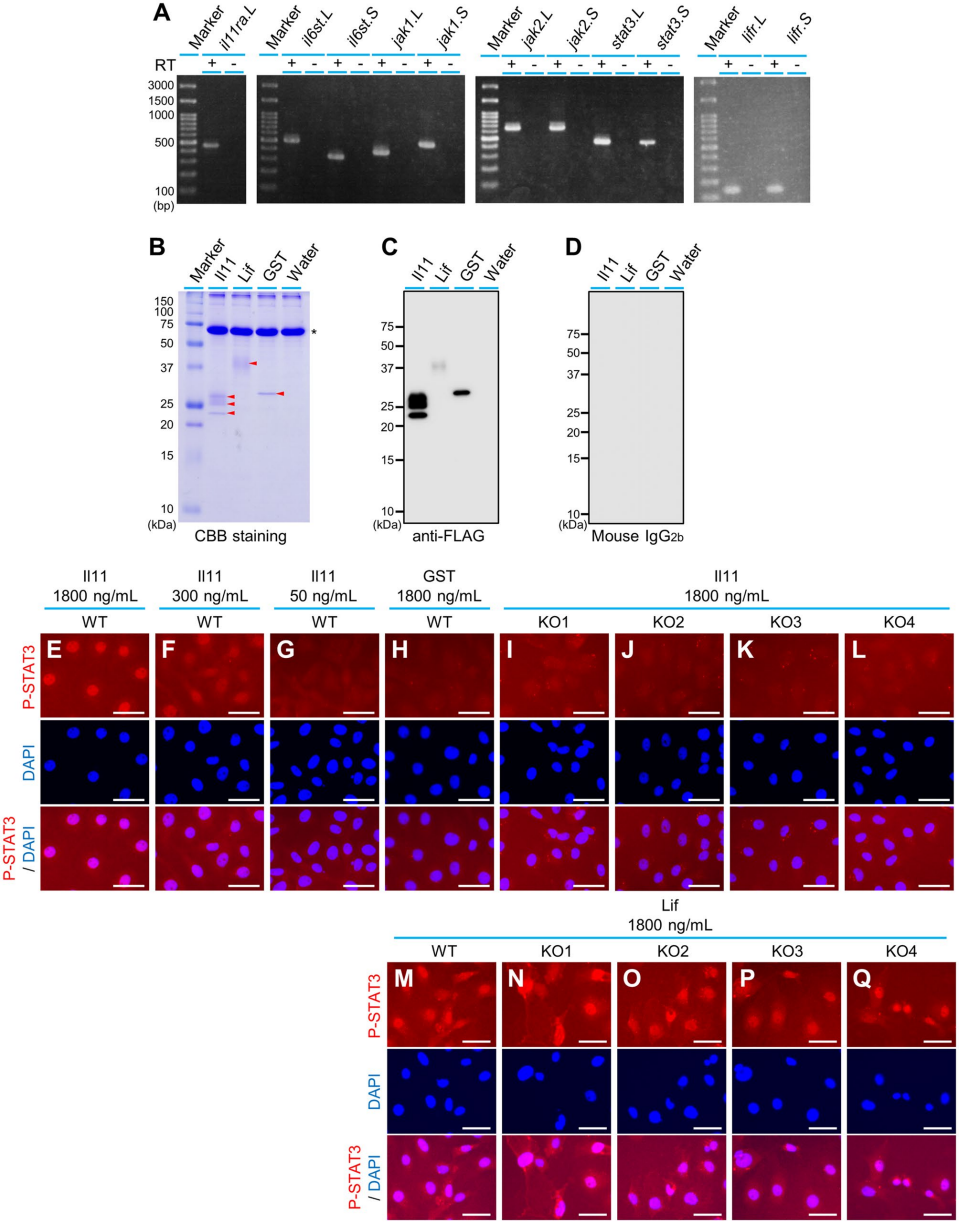
*X. laevis* Il11ra.L functions as an Il11 receptor component. (**A**) Gene expression involved in Il11 signaling in the XTC-YF cell line. (**B**–**D**) Oocyte culture supernatants were subjected to SDS-PAGE, followed by (**B**) CBB staining and (**C**) Western blotting using anti-FLAG antibody and (**D**) mouse IgG_2b_ isotype control. Size of the synthesized proteins estimated by their amino acid sequences was: Il11, 22.2 kDa; Lif, 25.5 kDa; and GST, 28.4 kDa. Arrowheads in (**B**) indicate the synthesized proteins. The 66.4-kDa bands (asterisk) in (**B**) the CBB staining represent BSA contained in the culture medium. (**E**–**Q**) Detection of XTC-YF P-Stat3 nuclear translocation by immunostaining. (Top) P-Stat3 signal is shown in red. (Middle) Nuclei were stained with DAPI. (Bottom) Merged images of P-Stat3 and DAPI staining. Data shown are representative of 3 independent experiments (except the experimental condition of Il11 50 ng/mL; n = 2). Scale bars: 50 µm. Raw images of the gel and blots are shown in Fig. S8.

We utilized the XTC-YF cultured cell line^20^, derived from *X. laevis* tadpole. In mammals, signal transduction to Stat3 phosphorylation in response to Il11 occurs via the Il11ra and Gp130 (also known as IL6ST) complex, JAK and STAT3^13–18^. The corresponding genes in *X. laevis* are *il11ra.L*, *il6st.L*, *il6st.S*, *jak1.L*, *jak1.S*, *jak2.L*, *jak2.S*, *stat3.S,* and *stat3.L*, and reverse transcription-polymerase chain reaction (PCR) confirmed that these genes are expressed in XTC-YF (Fig. 1A, S1A). Next, we established 4 lines of *il11ra.L* KO XTC-YF by transfection of *cas9* mRNA and 2 types of guide RNAs (#1 or #2) having different *il11ra.L* target sites (Fig. S1B, C). We also synthesized recombinant *X. laevis* Il11 protein by injecting *X. laevis il11* mRNA into *X. laevis* oocytes. For a negative Il11 treatment control, we synthesized recombinant glutathione S-transferase (GST). The mRNAs have a signal peptide sequence and a *3* × *flag* tag sequence (Fig. S2). Oocytes injected with these mRNAs were cultured, and culture supernatants containing the secreted proteins were collected. Protein syntheses were confirmed by sodium dodecyl sulfate–polyacrylamide gel electrophoresis (SDS-PAGE) followed by Coomassie Brilliant Blue (CBB) staining and Western blotting with anti-FLAG antibody (Fig. 1B–D). Both CBB staining and Western blotting detected 3 bands with molecular weights of approximately 23, 26 and 28 kDa in the culture supernatants of oocytes expressing recombinant Il11 (Fig. 1B, C), which were considered to be differently modified forms of Il11.

We added the oocyte culture supernatants to the medium of the XTC-YF wild-type (WT) and XTC-YF *il11ra.L* KO cell lines, and performed immunostaining using an anti-P-Stat3 antibody. Nuclear translocation of P-Stat3 was detected when Il11 was added to the WT cells (Fig. 1E–G). The signal intensity increased depending on the concentration of recombinant Il11 added to the WT cell culture medium (Fig. 1E–G), whereas no significant signal was detected when recombinant GST was added (Fig. 1H), strongly suggesting that Il11 treatment induced the nuclear translocation of P-Stat3. On the other hand, when recombinant Il11 was added to the KO cell culture medium, no significant nuclear translocation of P-Stat3 was detected (Fig. 1I–L). To confirm that the KO cells retain responsivity to another ligand that elicits nuclear P-Stat3 accumulation, we performed the same experiments using leukemia inhibitory factor (Lif). Lif is also a member of the Il6 cytokine family, and binds to the Lif receptor complex comprising the Lif receptor subunit (Lifr) and Gp130, which elicits Stat3 phosphorylation and nuclear translocation^21^. *X. laevis* has 2 *lifr* genes, *lifr.L* and *lifr.S*, and we confirmed their expression on XTC-YF (Fig. 1A, S1A). We synthesized recombinant *X. laevis* Lif.L that was Flag-tagged at the C-terminal by injecting the appropriate mRNAs into oocytes (Fig. S2). Protein synthesis was confirmed by CBB staining and Western blotting (Fig. 1B–D). The synthesized Lif-Flag protein had 224 amino acid residues whose predicted molecular size was 25.5 kDa. We detected a smeared band at 35–40 kDa (Fig. 1B, C), probably due to glycosylation because murine Lif is expressed in highly and variably glycosylated forms^22,23^. We administered the recombinant Lif to the WT and KO cells, and detected nuclear translocation of P-Stat3 in all of them (Fig. 1M–Q), indicating that the KO cells retain the functional signaling machinery required for nuclear translocation of P-Stat3. These results strongly suggest that *X. laevis* Il11ra.L functions as a Il11 receptor component in XTC-YF.

### *X. laevis il11ra.L* is required for tadpole tail regeneration

We next investigated whether *X. laevis* Il11ra.L also acts as an Il11 receptor component in tadpole tail regeneration. Knockdown (KD) of *il11* impairs tail regeneration in tadpoles^12^. Thus, we examined whether KD of *il11ra.L* also impairs tadpole tail regeneration.

**Figure 2:**
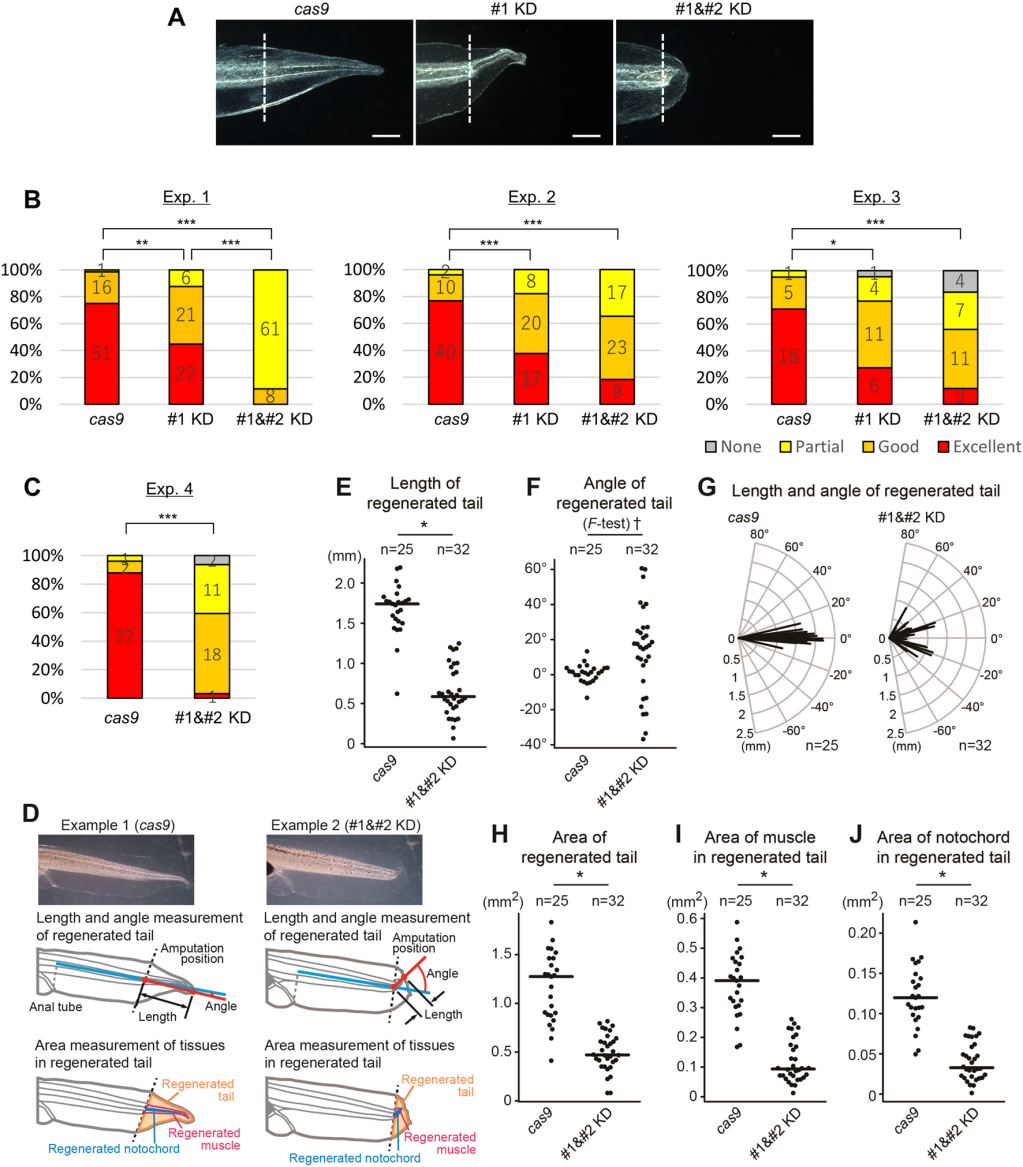
*il11ra.L* is required for *X. laevis* tadpole tail regeneration. (**A**) Typical examples of regenerated tails at 7 dpa of tadpoles injected with either (left) *cas9* mRNA only, (center) *cas9* mRNA and guide RNA #1, or (right) *cas9* mRNA, and guide RNA #1 and #2. Dashed lines indicate the amputation site. Scale bars: 500 µm. (**B**) Regeneration outcomes of *il11ra.L* KD tadpoles. Tadpoles at 7 dpa were classified into 4 groups depending on their tail regeneration: Excellent, completely regenerated with all tissues; Good, with all tissues but short or bent tail; Partial, with tissue(s) defect; None, no regeneration. Numbers shown in the bar chart are numbers of tadpoles classified into each group. Statistical significance was assessed by Fisher’s exact test with the Benjamini–Hochberg method, *P < 0.05, **P < 0.01, ***P < 0.001. (**C**–**J**) Measurement of regenerated tails. (**C**) Regeneration outcomes of the KD experiment used for measurement. The same classification criteria as in (**B**) were used. *cas9* group, n = 25; #1&#2 KD group, n = 32. ***P < 0.001 by Fisher’s exact test. (**D**) Schematic figure of the measurement. (**E**) Length of the regenerated tails. Bars indicate means. *P < 1 × 10^–10^ by Welch's *t*-test. (**F**) Axis angle of the regenerated tails. The dispersion in the 2 groups was compared by *F*-test, †*P* < 1 × 10^–10^. (**G**) Combined plot of length and axis angle of the regenerated tails. (**H**–**J**) Measured area of (**H**) whole regenerated tail, (**I**) muscle and (**J**) notochord in the regenerated tail. Bars indicate means. *P < 1 × 10^–9^ by Welch's *t*-test.

We set up 3 experimental groups; (1) embryos injected with *cas9* mRNA and *il11ra.L* guide RNA #1 (#1 KD); (2) embryos injected with *cas9* mRNA, and both guide RNAs #1 and #2 to improve KD efficiency (#1&#2 KD); and (3) a control group in which the embryos were injected with only *cas9* mRNA. We selected normally developed tadpoles 4 days post fertilization (4 dpf; stage 41) and amputated their tails. A heteroduplex mobility assay^24,25^ using amputated tails confirmed that the gene editing was successful (Fig. S3). Tail regeneration was evaluated in the tadpoles at 7 days post amputation (dpa). Tail regenerative abilities were significantly reduced in both the #1 KD and #1&#2 KD groups compared with the control group (Fig. 2A, B). The #1&#2 KD group showed or tend to show a significant decrease in tail regenerative ability compared with the #1 KD group. To assess the regeneration defects in *il11ra.L* KD tadpoles, we measured several parameters of the regenerated tails of *cas9* and #1&#2 KD tadpoles (Fig. 2C–J). The regenerated tails in the KD group were significantly shorter than those in the *cas9* group (Fig. 2E). In contrast to the axis angle of the regenerated tails in the *cas9* group, which ranged from -20 to 20 degrees, the axis angle in the KD group ranged from -40 to 60 degrees, with the dispersion of the axis angle of the KD group being significantly larger than that in the *cas9* group (Fig. 2F). Thus, the regenerated tails in the KD group were significantly shorter and had a more bent axis compared with those in the *cas9* group (Fig. 2G). We also assessed tissue regeneration by measuring the side view area of the whole regenerated tail, the muscles and the notochord in the regenerated tails, and found that the regenerated tails in the KD group were significantly smaller, and had smaller muscles and notochord (Fig. 2H–J), indicating that the *il11ra.L* KD affected muscle and notochord regeneration. These results strongly suggested that *il11ra.L* is necessary for tadpole tail regeneration, and also suggesting that *X. laevis* Il11ra.L also functions as an Il11 receptor component in tadpole tail regeneration. The ratios of normally developed tadpoles, and of surviving tadpoles after tail amputation did not differ significantly among the *cas9*, #1 KD, and #1&#2 KD groups (Table S1), suggesting that while *il11ra.L* is dispensable for development and survival after tail amputation, it has a crucial role in tail regeneration, like *il11*^12^.

To assess whether *il11ra.L* KD affects other early signaling events of regeneration, we compared gene expression profile changes after tail amputation between the *cas9* and *il11ra.L* KD groups. Tail stumps of tadpoles at 0 and 24 h post amputation (hpa) in the *cas9* and *il11ra.L* KD groups were sampled, and RNA-sequencing (RNA-seq) was performed. We screened those genes that were significantly more upregulated in the 24 hpa *cas9* sample compared with the others; i.e. genes for which 1) expression did not differ significantly between the *cas9* 0 hpa samples and the KD 0 hpa samples, suggesting that their developmental expression was not affected by *il11ra.L* KD, 2) expression was higher in *cas9* 24 hpa samples than in *cas9* 0 hpa samples, suggesting that their expression was upregulated by tail amputation, and 3) expression did not differ significantly between 0 and 24 hpa of the KD samples, suggesting that the upregulation by tail amputation was abolished in the *il11ra.L* KD tadpoles. The screening identified 6 genes exhibiting the described expression pattern (familywise error rate < 0.05; Table S2, Fig. S4). Notably, *wnt5a.L* was among the 6 genes. In *X. laevis* tadpole tail regeneration, *wnt5a* expression is upregulated at tail stumps after amputation^26–28^, and administration of Wnt5a to the tail trunk induces an ectopic tail^28^, suggesting function of induce tail regeneration. The RNA-seq suggested that induction of *wnt5a* expression after tail amputation requires *il11ra.L*-mediated signaling, emphasizing the important role of Il11 signaling in the early events of regeneration^12^.

### *il11ra.L* is widely and constitutively expressed in intact tadpoles and regenerating tails

We next performed in situ hybridization studies to investigate the localization of *il11ra.L*-expressing cells in tissue sections of intact tadpoles and regenerating tails. We analyzed WT intact 4 dpf tadpoles, and WT 1, 3, 5 dpa tadpoles that were amputated at 4 dpf.

**Figure 3:**
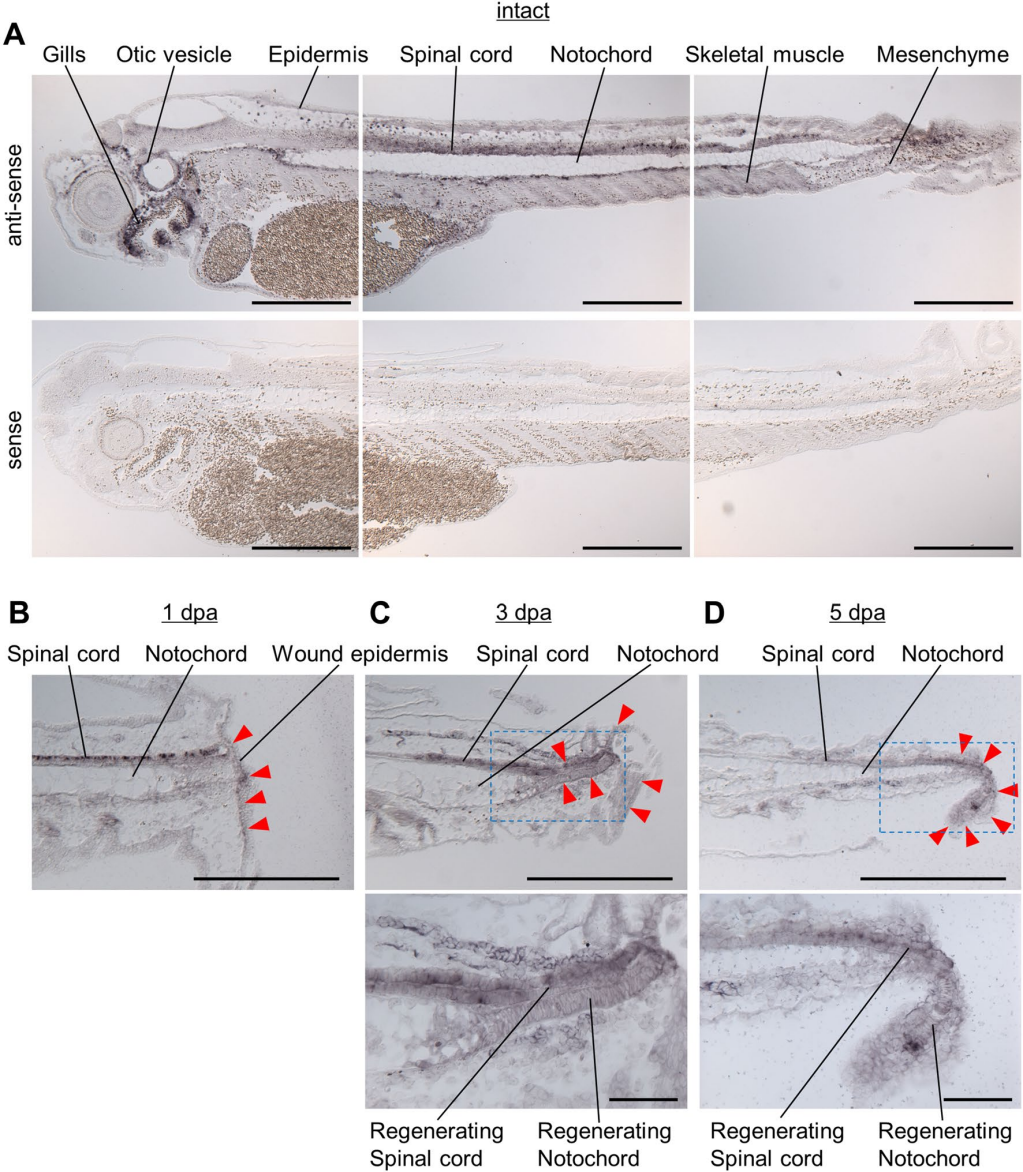
*il11ra.L* is widely and constitutively expressed in the intact tadpole and regenerating tail. (**A**) In situ hybridization of *il11ra.L* on a section of intact tadpole (upper) with an antisense probe, and (lower) with a sense probe. Anterior is left and dorsal is up. Scale bars: 500 µm. n = 4. (**B**–**D**) In situ hybridization of *il11ra.L* in a section of a regenerating tail. Anterior is left and dorsal is up. Arrowheads indicate notable signals. Scale bars: 500 µm. High magnification images of boxed areas in (**C**) and (**D**) are shown below. Scale bars: 100 µm. 1 dpa, n = 5; 3 dpa, n = 4; 5 dpa, n = 5.

In the whole bodies of intact tadpoles, *il11ra.L* expression was detected in many, but not all cells of many tissues, including the spinal cord, notochord, skeletal muscle, epidermis, and mesenchyme (Fig. 3A), indicating that *il11ra.L* is expressed constitutively in 4 dpf tadpoles. In the tails of 1 dpa tadpoles, *il11ra.L* expression was observed in the uninjured site tissues, as well as throughout the wound epidermis (Fig. 3B). In tails of 3 dpa and 5 dpa tadpoles, expression was observed in the uninjured tissue, and the expression pattern in regenerated tissues at the anterior side of the regenerating tail was similar to that in the tail tissues anterior to the amputation (Fig. 3C, D). In addition, stronger signal was observed throughout the regenerating notochord and spinal cord at the tip of the regenerating tail (Fig. 3C, D). These observations correspond to those of the previous quantitative analysis of *il11ra.L* expression during tail regeneration in which *il11ra.L* expression was detected at 0 hpa and modestly increased thereafter^12^, as well as to the result of single cell RNA-seq of intact/regenerating tails^8^ in which *il11ra.L* expression was detected in neurons, spinal cord progenitors, floor plate, notochord, myotome, epidermis, and mesenchyme (Fig.S5).

### *il11ra.L* KO precursors can also contribute to form regenerated tails

We previously reported that *il11* is necessary for tail regeneration, and forced expression of *il11* induces the expression of undifferentiated precursor marker genes, indicating that *il11* plays a key role in inducting and maintaining undifferentiated precursors for tail regeneration^12^. This function of *il11* is likely mediated via the Il11 receptor that receives Il11 secreted in response to tail amputation. Therefore, we next investigated whether Il11 is received by tissue stem cells or precursors to generate undifferentiated cells (“Direct model”; Il11 directly activates stem cells or precursors), or if it is received by some other cells that subsequently activate tissue stem cells or precursors to generate undifferentiated cells (“Indirect model”; Il11 triggers downstream events that activate stem cells or precursors).

We performed the following experiments (Fig. S6). We generated *il11ra.L* KD tadpoles by injecting mRNA and guide RNA #1 into 1-cell stage embryos (same as in Fig. 2). The *il11ra.L* KD embryos were assumed to have developed into mosaics comprising 3 cell types; WT, heterozygously KO (“HT”), and homozygously KO (referred to as “KO”) cells. *il11ra.L* KD tadpoles had a normal morphology and survival rate (Table S1), and the tail cells were mosaics of the above 3 cell types. The tissue stem cells or progenitors that contribute to form regenerated tail tissues were also assumed to be mosaics. Tail amputation triggers Il11 expression and induces undifferentiated cells. If Il11 directly activates stem cells or progenitors upon tail amputation (“Direct model”), *il11ra.L* KO progenitors are not activated, and these KO cells do not contribute to form the regenerated tail; thus, regenerated tail tissues mainly comprise *il11ra.L* WT or HT cells, but not KO cells. On the other hand, if Il11 indirectly activates progenitors via some Il11 receptor-expressing cells (“Indirect model”), *il11ra.L* KO progenitors are activated like WT or HT cells, and these KO cells contribute to form the regenerated tail. To test this, we generated *il11ra.L* KD tadpoles and amputated their tails at 4 dpf. Amputated tails were sampled as “developmental tails”. Amputated tadpoles were maintained for 7 days and regenerated tails were sampled as “regenerated tails”. We extracted genomic DNA from these tail samples, and compared the mutation (insertion/deletion) ratio at the guide RNA #1 target site of the *il11ra.L* genomic locus of developmental tails with that of regenerated tails in the same individual. If *il11ra.L* KO cells do not contribute to form regenerated tails, the mutation ratio of the regenerated tail would be lower than that of the developmental tail, which contains KO cells.

**Figure 4:**
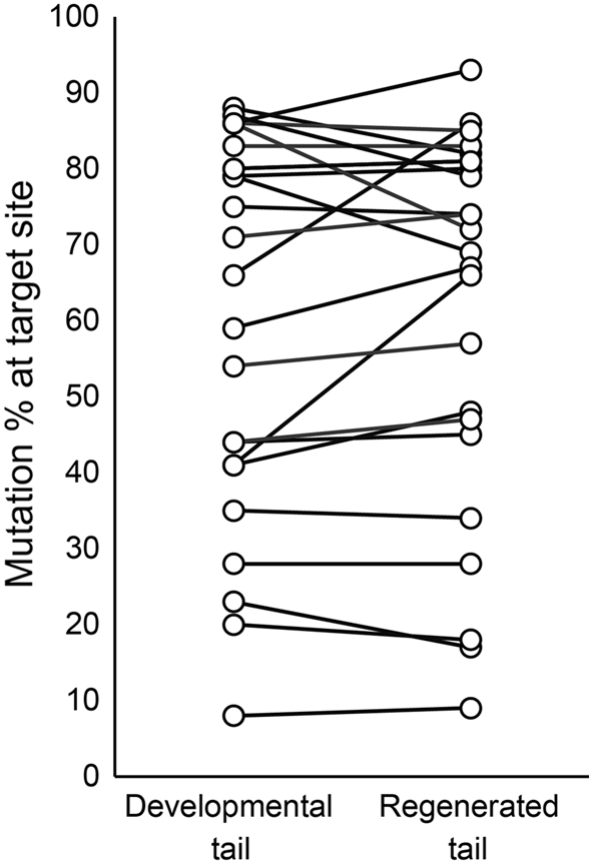
*il11ra.L* KO cells can contribute to form the regenerated tail. Estimated mutation ratio at the guide RNA #1 target site of the developmental and regenerated tail of each individual is shown. Linked points represent the same individual. n = 24. *P* = 0.44, paired *t*-test. Representative result of 2 experiments is shown. The result of the second experiment is shown in Fig. S7.

The mutation ratio of the developmental tails was not significantly different from that of the regenerated tails (Fig. 4), suggesting that some *il11ra.L* KO precursors contribute to form regenerated tails and that these precursors do not require cell autonomous *il11ra.L* expression for tail regeneration. Although the mode of Il11 function might differ according to the tissue type, we detected no significant decrease in the mutation ratios, supporting the idea that at least the tissues comprising a large portion of the regenerated tails are formed in a way depicted by the “Indirect model”.

## Discussion

The present findings demonstrated that *il11ra.L,* whose function as an Il11 receptor has not been verified experimentally in *X. laevis*, is required for the nuclear localization of P-Stat3 in response to Il11 in cultured *X. laevis* cells (Fig. 1), strongly suggesting that *il11ra.L* encodes an Il11 receptor complex component. This finding also suggests that the receptor complex containing Il11ra.L is the only or at least the major Il11 receptor mediating Il11 signals to phosphorylate Stat3 in cultured *X. laevis* cells. In the assay, we used recombinant proteins at a concentration of 1800 ng/mL, which is thought to be well above the endogenous levels of most ligands. The P-Stat3 nuclear localization signals were weaker with 300 ng/mL Il11, and hardly detected with 50 ng/mL Il11 (Fig. 1E–G). For example, human umbilical vein endothelial cells^29^ and rat microglia^30^ respond to 50 ng/mL Il11. Possible reasons for XTC-YF requiring a higher concentration of Il11 to respond in our experimental conditions are as follows: 1) XTC-YF is less sensitive to Il11, which might be due to the relatively low expression of *il11ra.L* (Fig. S1A), and/or 2) not all forms of the recombinant Il11 that we detected (Fig. 1B, C) were functional. Even in this situation, we observed clear differences in the response to Il11 between WT and *il11ra.L* KO cells, strongly suggesting that *il11ra.L* is essential for Il11 signaling.

*il11ra.L* KD did not affect the ratios of normally developed tadpoles and surviving tadpoles after tail amputation (Table S1), suggesting that *il11ra.L* is dispensable for normal development and wound healing after tail amputation. On the other hand, *il11ra.L* KD significantly impaired tail regeneration (Fig. 2), indicating a specific role of *il11ra.L* in tail regeneration. These phenotypes are similar to *il11* KD^12^, supporting the idea that *il11ra.L* is the only or at least a major mediator of Il11 signaling in tadpole tail regeneration. We also observed that *il11ra.L* KD significantly affected the expression of 6 genes that are induced by tail amputation, including *wnt5a* (Table S2, Fig. S4)*.* There are several reports of *wnt5a* function in development, particularly elongation/outgrowth of body parts in several species^31–34^, suggesting that one of the *wnt5a* functions in tail regeneration is elicited during regenerative outgrowth^26–28^. The results suggested that *wnt5a* induction after tail amputation requires Il11 signaling. It is plausible that a pivotal function of Il11 signaling in regeneration is controlling *wnt5a* expression and thus regenerative outgrowth, because KD of *il11*^12^ or *il11ra.L* (Fig. 2E) shortened the regenerated tails. Although there are no reports on the function of the other 5 upregulated genes in tail regeneration of *X. laevis*, it is expected that these genes also have pivotal roles in regeneration. Our results indicated that Il11 signaling mediated by Il11ra.L has a specific and indispensable role in tail regeneration.

Il11 signaling is mediated by Il11ra.L, indicating that *il11ra.L*-expressing cells play pivotal roles in tail regeneration. We observed constitutive and widespread expression of *il11ra.L* in most tissues in intact tadpoles and regenerating tails (Fig. 3). Tail amputation triggers *il11* expression near the wound stump^11,12^, suggesting its specific and local role in regeneration. The constitutive and widespread expression of *il11ra.L* might be beneficial for the response to accidental (i.e., temporally and spatially unpredictable) wounding; *il11ra.L*-expressing cells throughout the body are ready to respond to Il11 secreted from wounds in any body part. It is unclear, however, whether all or only some *il11ra.L*-expressing cells have the potential to respond for regeneration.

Although *il11ra.L* is required for tail regeneration (Fig. 2), we detected no significant differences in the mutation rates of developmental and regenerated tails of *il11ra.L* KD tadpoles (Fig. 4). These results support the existence of a tissue(s) in which Il11 functions via *il11ra.L* expressing cells in a non-cell autonomous manner; the functions of Il11 are elicited by factors expressed in cells receiving Il11. Some *il11ra.L*-expressing cells probably control tail regeneration depending on Il11 secretion via downstream genes of *il11ra.L*. It remains unclear, however, whether the *il11ra.L* KO cell contribution is common in all tail tissues or differs according to the tissue type. To address this question, a transgenic frog line in which all *il11ra.L* KO cells are labeled must be established; at least 1 generation of this long-generation (more than 18 months) animal is required.

In *X. laevis*, both *il11*^12^ and *il11ra.L* (Fig. 2) are required for tail regeneration. *il11* is assumed to be required for heart regeneration in fish, because *il11* expression is observed in the regenerating heart, and Stat3 inhibition impairs heart regeneration^35^. In mouse, on the other hand, *il11* enhances fibrosis in the heart, which causes mechanical dysfunction, and genetic deletion of *Il11ra1* reduces the fibrosis^36^. Mouse heart has full regenerative capacity at postnatal days 1–6 (P1-6), but this capacity is lost by P7 with the development of fibrosis after apical resection^37^. Whereas induction of *il11* expression in organ regeneration or wound healing is conserved across animal species, it is possible that the downstream events of Il11, which are elicited via *il11ra* expressing cells, differ between regeneration-capable or incapable species/developmental stages/tissues. Our study highlights the pivotal role of *il11ra* in organ regeneration and provides a basis for future studies of the role of Il11 in organ regenerative ability.

## Materials and methods

### Animals

*Xenopus laevis* adults were purchased from domestic breeders (Hamamatsu Seibutsu Kyozai, Shizuoka, Japan; Watanabe Zoshoku, Hyogo, Japan) and maintained in fresh water at 20 °C. Animal experiments were carried out in accordance with the Guidelines for Proper Conduct of Animal Experiments of Science Council of Japan. The protocols of experiments using living modified organisms in this study were approved by the Committee on living modified organisms experiments of the Graduate School of Science at the University of Tokyo (DNA Exp. 17–3). Experiments and data analysis procedures were performed in compliance with the ARRIVE guidelines (https://arriveguidelines.org).

### Cultured cells

The XTC-YF cell line derived from *X. laevis* tadpoles^20^ (RIKEN BioResource Research Center, Ibaraki, Japan) was maintained in 0.5 × L-15 medium for cultured cells (0.5 × Leibovitz's L-15 medium (Merck, Darmstadt, Germany), 10% fetal bovine serum [FBS; BioWest, Nuaillé, France], 2 mM L-glutamine [Wako, Osaka, Japan], and 1% penicillin–streptomycin mixture [Nacalai Tesque, Kyoto, Japan]) at 25 °C.

### Reverse transcription-PCR (RT-PCR) and quantitative RT-PCR (qRT-PCR) of XTC-YF

For RT-PCR, total RNA was extracted from XTC-YF using TRIzol Reagent (Thermo Fisher Scientific, Waltham, MA). Reverse transcription was performed using a PrimeScript RT reagent Kit with gDNA Eraser (Takara, Shiga, Japan). A group without reverse transcription (RT-) was also prepared as a control. Sequences of primers used are shown in Table S3.

For qRT-PCR, total RNA was extracted from 3 lots of XTC-YF using RNeasy Mini Kit (QIAGEN, Hilden, Germany). Reverse transcription was performed using a PrimeScript RT reagent Kit with gDNA Eraser. A group without reverse transcription (RT-) was also prepared as a control. Sequences of primers used are shown in Table S4. Realtime-PCR was performed with TB Green Premix Ex Taq II (Tli RNaseH Plus) (Takara) and LightCycler 480 (Roche, Basel, Switzerland). The threshold cycle was calculated using the 2nd derivative maximum method.

### Establishment of XTC-YF *il11ra.L* knockout cell lines

The CRISPR/*cas9* system was used. The *il11ra.L* guide RNAs were designed with CRISPRdirect^38^. The target sequences were as follows: #1, GGTGAGAAGCACAATCACC[CGG]; and #2, GGGAGCAGCGTCTCAACAC[TGG] (sequences in brackets indicate the protospacer adjacent motif). The target sequences were inserted into a DR274 plasmid^39^ and guide RNAs were synthesized using an AmpliScribe T7-Flash Transcription Kit (Lucigen, Middleton, WI) before purification with an RNeasy Mini Kit. For *cas9* mRNA synthesis, pXT7-hcas9^40^ (China Zebrafish Resource Center, Wuhan, China) was digested with *Xba* I (Takara) and then mRNA was synthesized using an mMESSAGE mMACHINE T7 ULTRA Kit (Thermo Fisher Scientific, Waltham, MA) and purified with an RNeasy Mini Kit (QIAGEN).

*il11ra.L* guide RNA #1 or #2 and *cas9* mRNA were transfected into XTC-YF cells using Lipofectamine LTX Reagent (Thermo Fisher Scientific). After cloning by limiting dilution, cells of cloned cultures were suspended in 50 mM NaOH at 98 °C for 10 min, and neutralized with 1/10 vo1ume of 1 M Tris–HCl pH 7.5 to obtain genomic DNA extract. Regions including the guide RNA target site were amplified by PCR with primers (sequences are shown in Table S5), and Sanger sequencing with the forward primers was performed. The sequence data were analyzed with CRISP-ID^41^. Colonies with only 1 or 2 types of base insertions/deletions with frameshifts were determined to be derived from 1 cell with knockout of both alleles of *il11ra.L*. We established 4 lines of *il11ra.L* KO XTC-YF (2 lines [KO1 and KO2] with guide RNA #1, and 2 lines [KO3 and KO4] with guide RNA #2). The obtained sequences are shown in Fig. S1B, C.

### Synthesis of recombinant *Xenopus laevis* Il11

Recombinant protein was synthesized by mRNA injection into *X. laevis* oocytes. The construction procedures are shown in Fig. S2. The *il11.L* sequence (AB933563) was amplified and the *3* × *flag* tag sequence was inserted between the *il11.L* signal peptide and mature peptide sequences (Fig. S2). The resultant N-terminal *3* × *flag*-tagged *il11.L* sequence was inserted into a pXT7 plasmid. mRNA was synthesized as described above. For a negative Il11 treatment control, we synthesized recombinant GST, which is hydrophilic and has a molecular weight close to that of Il11. For mRNA of GST, the *il11.L* signal peptide sequence and *3* × *flag* tag sequence were added, and the mRNA was synthesized (Fig. S2). We also synthesized recombinant Lif; for *lif* mRNA, the *lif.L* sequence was obtained from Xenbase (http://www.xenbase.org/, RRID:SCR_003280) and amplified, the *flag* sequence was added to the C-terminus, and the mRNA was synthesized (Fig. S2).

Oocyte injection was performed essentially as described previously^42^. A part of the ovary was excised from an adult *X. laevis*. Oocyte follicles were removed manually with forceps in 1 × MBS (88 mM NaCl, 1 mM KCl, 0.7 mM CaCl_2_, 1 mM MgSO_4_, 5 mM HEPES [pH 7.8], and 2.5 mM NaHCO_3_), or enzymatically by treating oocytes in 0.5 × L-15 medium for oocytes (0.5 × Leibovitz's L-15 medium [Merck], 0.5 mg/mL bovine serum albumin [BSA; Wako], 2 mM L-glutamine [Wako], and 1% penicillin–streptomycin mixture [Nacalai Tesque]) supplemented with 25 mM HEPES [pH 7.6], 10 mM CaCl_2_, and 10 mg/ml collagenase (Nacalai Tesque) for 1 h. Oocytes were maintained overnight in 0.5 × L-15 medium for oocytes at 20 °C. We injected 50.6 nl of 1 ng/nl mRNA into oocytes. For the control group, an equal volume of RNase-free water was injected. Injected oocytes were maintained overnight and then the medium was changed. After 3 or 4 days, the oocyte culture supernatants containing the secreted protein were collected.

The supernatants were subjected to glycine-SDS-PAGE and concentrations of synthesized proteins were estimated on the basis of the intensity of CBB stained bands that were detected near the expected molecular size, and bands of a series of known amounts of BSA, using ImageJ. For Western blotting, mouse anti-DYKDDDDK (FLAG) antibody (Wako; clone 1E6) or mouse IgG2b isotype control (BioLegend, San Diego, CA) was used as the primary antibody, and Clean Blot™ IP Detection Reagent (HRP) (Thermo Fisher Scientific) was used as the secondary antibody. The signal was detected by chemiluminescence using ECL select (Cytiva, Tokyo, Japan), and ImageQuant LAS 4000 (Cytiva).

### Il11 administration to XTC-YF and immunohistochemistry of phosphorylated STAT3

XTC-YF wild-type and *il11ra.L* knock-out cell lines were seeded on 96-well plates. After serum starvation in a serum-free medium (0.5 × L-15 medium for cultured cells without FBS) for 4 h, the oocyte culture supernatant containing recombinant proteins was added to the medium. The final concentrations were 1800 ng/ml, 300 ng/ml, and 50 ng/ml for Il11 and GST; and 1800 ng/ml for Lif. After 20 min, cells were fixed with 4% paraformaldehyde/PBS and then permeabilized with cooled methanol. Anti-phospho-STAT3 (Tyr705) antibody (Cell Signaling Technology, Beverly, MA, #9138S) and Alexa Fluor 555-conjugated anti-mouse IgG goat antibody (Invitrogen, Carlsbad, CA, A-21424) were used to stain phospho-STAT3. Nuclei were stained with 1 µg/ml DAPI. Cells were observed using a fluorescence microscope BZ-X800 (KEYENCE, Osaka, Japan).

### Generation of *il11ra.L* KD tadpoles and evaluation of tail regenerative ability

Guide RNAs and *cas9* mRNA prepared as described above were used. Injection was performed essentially as described previously^12,43,44^. Fertilized eggs were dejellied with 3% cysteine and divided into 3 groups; 18.4 nl of (1) *cas9* mRNA (700 ng/µl), (2) *cas9* mRNA (700 ng/µl) and *il11ra.L* guide RNA #1 (40 ng/µl), (3) *cas9* mRNA (700 ng/µl), *il11ra.L* guide RNA #1 and #2 (40 ng/µl, respectively) were injected into the animal hemisphere of the egg. Injected embryos were maintained at 12 °C for 6 h and reared at 20 °C. Tadpoles of 4 dpf (stage 41) were anesthetized with 0.02% MS-222 (Sigma, St. Louis, MO) at 0.1 × Steinberg solution (5.8 mM NaCl, 67 µM KCl, 34 µM Ca(NO_3_)_2_, 83 µM MgSO_4_, 300 µM HEPES, pH7.4), and the tails were amputated. Tadpoles with amputated tails were kept in 0.1 × Steinberg solution 20 °C for 7 days, and then classified into 4 groups depending on tail regeneration (Excellent, Good, Partial, and None). Statistical significance was assessed by Fisher’s exact test with the Benjamini–Hochberg method^45^. For measuring the regenerated tails, we took photos of the regenerated tails of *cas9* and #1&#2 KD tadpoles at 7 dpa and the parameters shown in Fig. 2D were measured using Fiji^46^.

### Heteroduplex mobility assay (HMA)

A schematic of HMA is shown in Fig. S3A. Genomic DNA was extracted as described above. The regions including the guide RNA target site were amplified by PCR with primers (sequences are shown in Table S5). PCR products were subjected to electrophoresis using 3% agarose gel containing 45 mM Tris, 1 mM EDTA, and 45 mM boric acid.

### RNA-sequencing (RNA-seq) of tail stumps of il11ra.L KD tadpoles

*il11ra.L* KD (using gRNA #1 and #2) tadpoles and control tadpoles (no gRNAs) were generated as described above. The tails of 4 dpf tadpoles were amputated, and the amputated tail stumps (tail tips were removed) were collected as 0 hpa samples. Tadpoles with amputated tails were kept for 24 h and the tail stumps were collected as 24 hpa samples. We collected 3 lots of 17–24 tail stumps, and total RNA was extracted using the RNeasy Mini Kit. RNA-seq was performed at Genome-Lead Co., Ltd. (Takamatsu, Japan). The mRNA was isolated using KAPA mRNA Capture Kit (NIPPON Genetics, Tokyo, Japan), followed by production of cDNA libraries using MGIEasy RNA Directional Library Prep Set (MGI, Shenzhen, China). RNA-seq was performed using DNBSEQ-G400 RS (MGI) with the DNBSEQ G400RS High Throughput Sequencing Set, to generate approximately 4 × 10^7^ paired-end reads (150 bp × 2) from each cDNA library.

Obtained reads were mapped to *X. laevis* genome version 9.1 sequence (Xla.v91.repeatMasked.fa) using HISAT2 v2.1.0^47^, and the number of reads mapped to each gene was counted using FeatureCounts v2.0.1^48^ with a gene model (XL_9.1_v1.8.3.2.primaryTranscripts.gff3). The genome sequence and gene model were downloaded from Xenbase. Differential expression analysis was performed using baySeq package v2.26.0^49^.

### Estimation of mutation ratios of KD embryo tails

We generated *il11ra.L* KD tadpoles with guide RNA #1 as described above. Tails of 4 dpf tadpoles were amputated, and amputated tails were sampled as “developmental tails”. Amputated tadpoles were kept separately in 24 well plates (1 tadpole per well) for 7 days, and then regenerated tails were sampled as “regenerated tails”. Genomic DNA was extracted as described above. PCR with primers (sequences are shown in Table S6) was performed, followed by Sanger sequencing with the forward primer. Sequence data were subjected to ICE analysis^50^ (https://ice.synthego.com/) to estimate the mutation ratio.

### In situ hybridization of tissue sections

For RNA probe synthesis, a part of the *il11ra.L* coding sequence was amplified by PCR with primers (sequences are shown in Table S7) and cDNA from the thymi of *X. laevis* J strain tadpoles, and subcloned into a pGEM-T easy vector (Promega, Madison, WI). Digoxigenin-labeled RNA probes were synthesized with T7 or SP6 RNA polymerase (Roche) and DIG RNA Labeling Mix (Roche).

Tadpoles were obtained by artificial fertilization and maintained in 0.2% sea salt water at 20 °C. In situ hybridization was performed as described previously^12,51^ with several modifications. Wild-type intact 4 dpf tadpoles, and wild-type 1, 3, 5 dpa tadpoles which were amputated at 4 dpf, were fixed in MEMFA fixative (0.1 M MOPS (pH7.4), 2 mM EGTA (pH8.0), 1 mM MgSO_4_, and 10% formalin) at room temperature for 48 h. They were dehydrated with an ethanol and Clear Plus (Falma, Tokyo, Japan) series and embedded in Paraplast plus (Merck), and 7-µm thick sagittal sections were prepared. Sections were rehydrated, permeabilized with 20 µg/ml proteinase K (Roche) and 1% Triton X-100, followed by acetylation with 0.25% acetic anhydride. The digoxigenin-labeled probe were hybridized at 58 °C for 20 h, and washed with 0.3 × SSC (4.5 mM trisodium citrate, 45 mM NaCl, pH7.0). The slides were incubated with alkaline phosphatase conjugated anti-digoxigenin antibody (Roche). Signals were detected with NBT/BCIP stock solution (Roche) for 24 h. To bleach the endogenous melanin pigments, slides were placed in 0.5 × SSC containing 0.3% H_2_O_2_ under fluorescent light overnight. The slides were observed using a differential interference microscope.

## Supplementary Information

Supplementary Information.

## Data Availability

The RNA-seq data have been deposited in the DNA DataBank of Japan under accession code DRA012988. The datasets generated during the current study are available from the corresponding author upon reasonable request.
