# Supplementary material for: Xenopus laevis il11ra.L is an experimentally proven interleukin-11 receptor component that is required for tadpole tail regeneration
Source: Sci Rep. 2022 Feb 3;12:1903. doi: 10.1038/s41598-022-05954-w (PMC8814168; doi:10.1038/s41598-022-05954-w)
Supplement: Supplementary file 1 — Supplementary Information. [file 41598_2022_5954_MOESM1_ESM.pdf]

Supplementary Information

Xenopus laevis il11ra.L is an experimentally proven interleukin-11 receptor component that is required for tadpole tail regeneration.

Shunya Suzuki†, Kayo Sasaki†, Taro Fukazawa* & Takeo Kubo

Department of Biological Sciences, Graduate School of Science,

The University of Tokyo

†: equal contribution

*email: tfukazawa@bs.s.u-tokyo.ac.jp

A

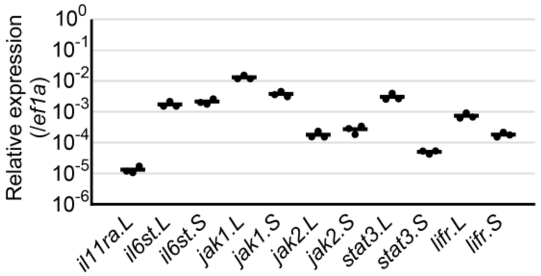

B

```
                             in/del              PAM     guide RNA #1 target site
X. laevis il-11ra.L                 ATCATGTCTTAGCCGGGT-----------------GATTGTGCTTCTCACCGTGAC
XTC-YF-il11ra.L-KO1           +17   ATCATGTCTTAGCCGGGTTTTAGATTTATGTCTTAGATTGTGCTTCTCACCGTGAC
XTC-YF-il11ra.L-KO2-1          Δ2   ATCATGTCTTAGCCGG-------------------GATTGTGCTTCTCACCGTGAC
XTC-YF-il11ra.L-KO2-2          Δ2   ATCATGTCTTAGCCGG-------------------GAGTGTGCTTCTCACCGTGAC
```

C

```
                             in/del              PAM guide RNA #2 target site
X. laevis il-11ra.L                 GCTTCTGGAAATCCAGTGTTGAGACGCTGCTCCCTACCAGATTCACTGCTTCCTAC
XTC-YF-il11ra.L-KO3           Δ13   GCTTCTGGAAATCC-------------TGCTCCCTACCAGATTCACTGCTTCCTAC
XTC-YF-il11ra.L-KO4            Δ1   GCTTCTGGAAATCCAGTG-TGAGACGCTGCTCCCTACCAGATTCACTGCTTCCTAC
```

Supplementary Fig. S1

Expression of Il11 signaling molecules in XTC-YF culture cell line and establishment of XTC-YF *il11ra.L* KO cell lines. (A) Expression levels of genes related to Il11 signaling measured by quantitative RT-PCR using 3 lots of RNA extracted from XTC-YF. Relative expression levels of the genes normalized by those of *ef1a* are shown in logarithmic scale. Bars indicate the mean value. (B and C) Genomic *il11ra.L* sequences of established XTC-YF *il11ra.L* KO cell lines. (B) KO1 and KO2 were established with guide RNA #1, (C) KO3 and KO4 were established with guide RNA #2. PAM, protospacer adjacent motif.

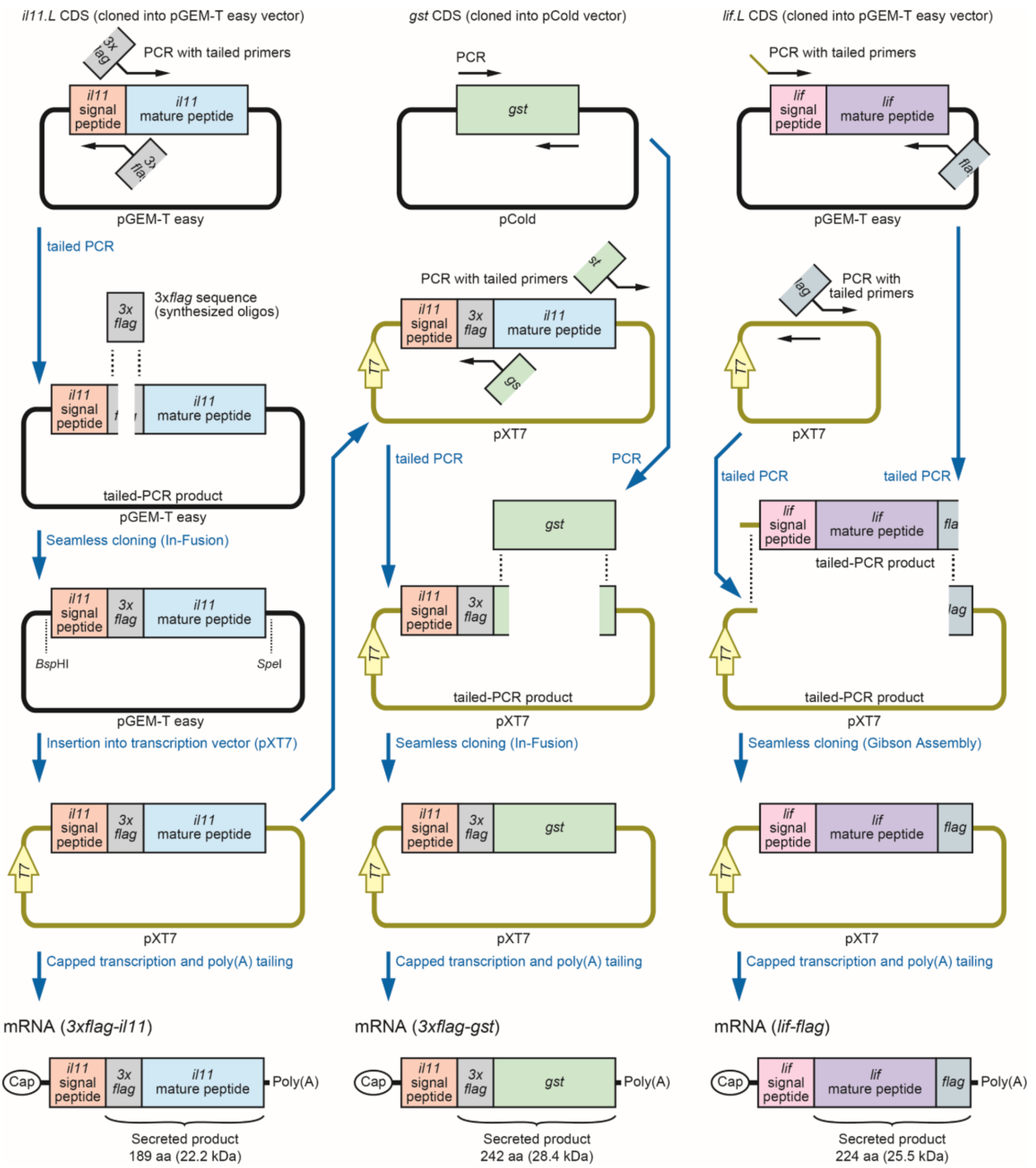

Supplementary Fig. S2
Construction of mRNAs of (left) N-terminal 3 × *flag*-tagged *il11.L*, (center) N-terminal 3 × *flag*-tagged *gst*, and (right) C-terminal *flag*-tagged *lif.L*.

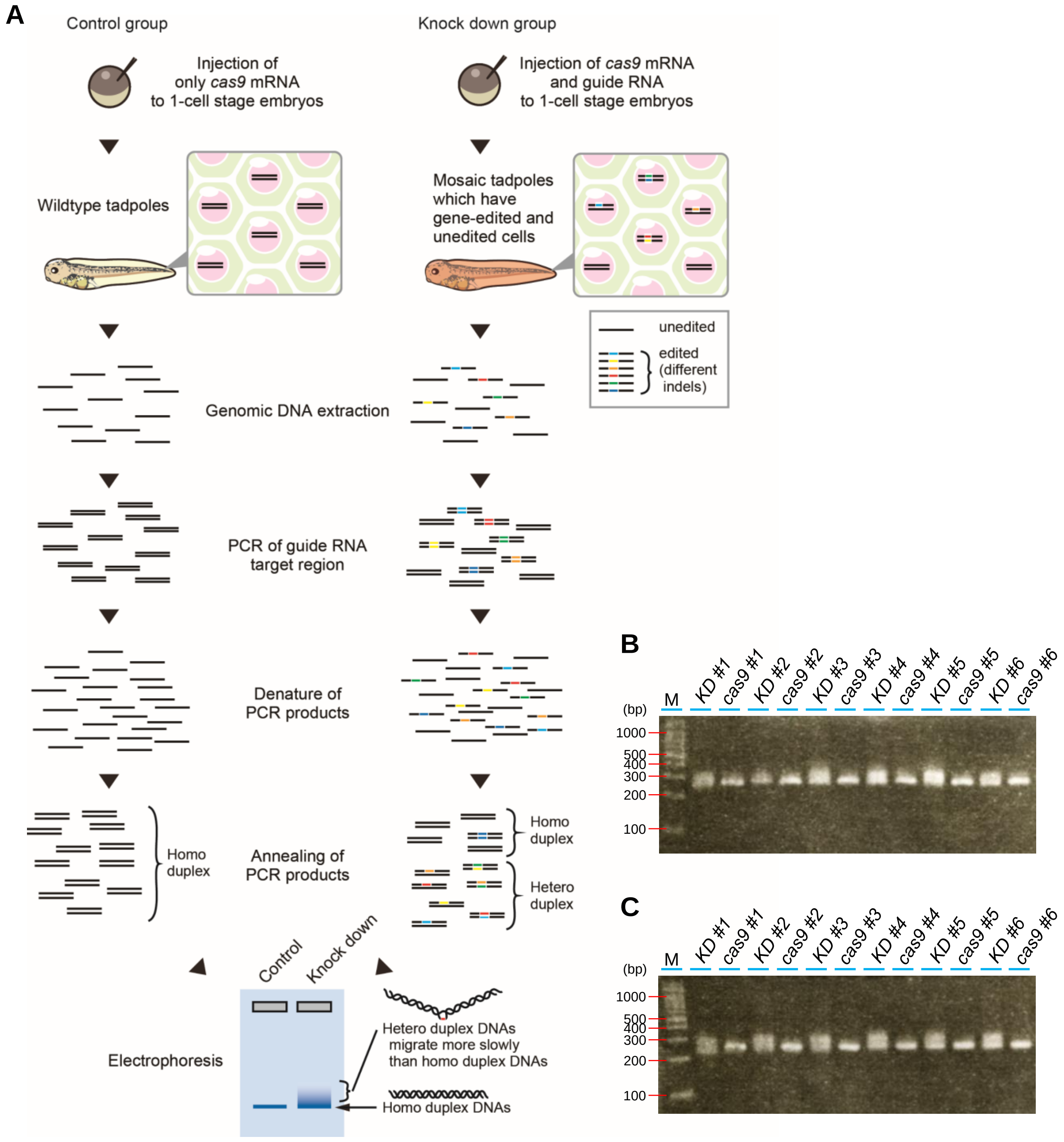

Supplementary Fig. S3

Heteroduplex mobility assay (HMA) for detection of gene editing. (A) Schematic figure of HMA. (B and C) HMA on amputated tails. Genomic loci including the target site of (B) guide RNA #1 or (C) #2 were amplified by PCR. Smeared bands representing multiple heteroduplexes are shown from the PCR products derived from embryos injected with guide RNA and *cas9* mRNA (KD), whereas single bands are shown from control embryos injected with only *cas9* mRNA (cas9). Numbers represent the serial numbers of samples. M, Marker lane. Full-length gel images are presented in Fig. S9.

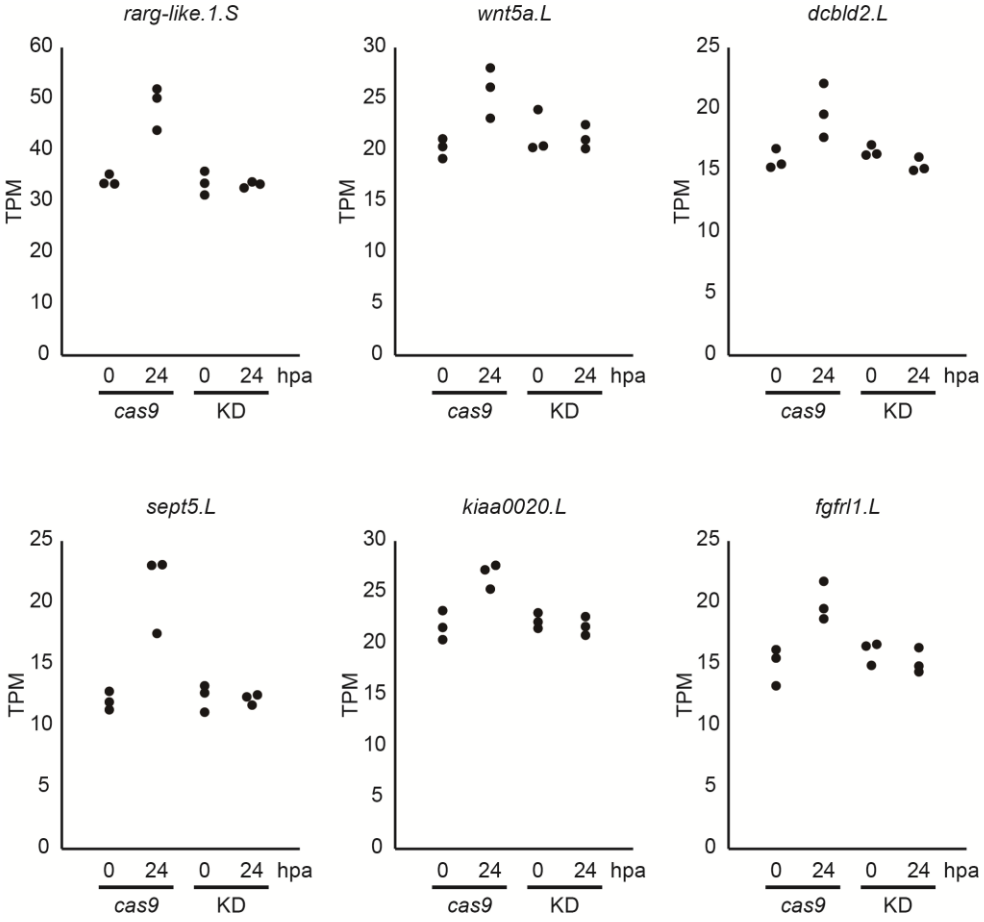

Supplementary Fig. S4

Expression of genes that are significantly upregulated in *cas9* 24 hpa tail stumps compared with *cas9* 0 hpa tail, and 0 and 24 hpa *il11ra.L* KD tail stumps. TPM, transcriptions per million.

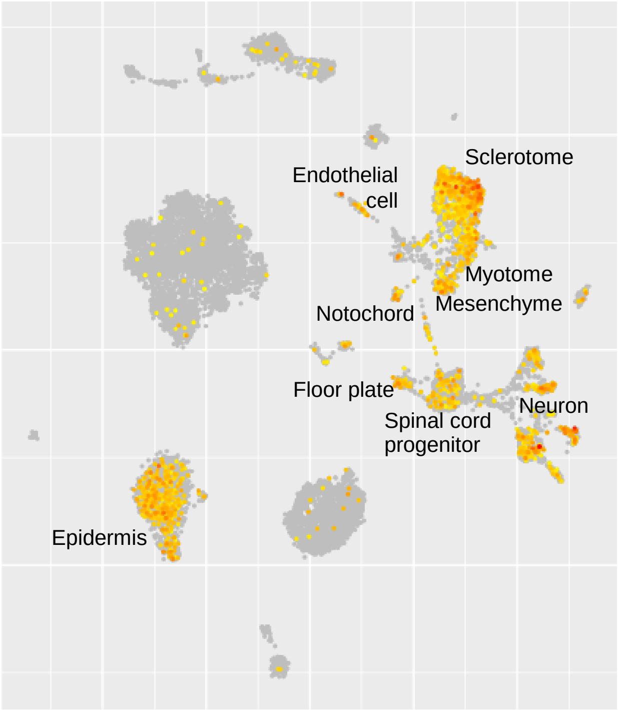

Supplementary Fig. S5

il11ra.L expression in single cell RNA-seq of regenerating tails reported by Aztekin *et al*.[1] is visualized with the web platform (Xenopus tail regeneration at single cell resolution; https://marionilab.cruk.cam.ac.uk/XenopusRegeneration/).

1. Aztekin, C. *et al.* Identification of a regeneration-organizing cell in the Xenopus tail. *Science* **364**, 653–658 (2019).

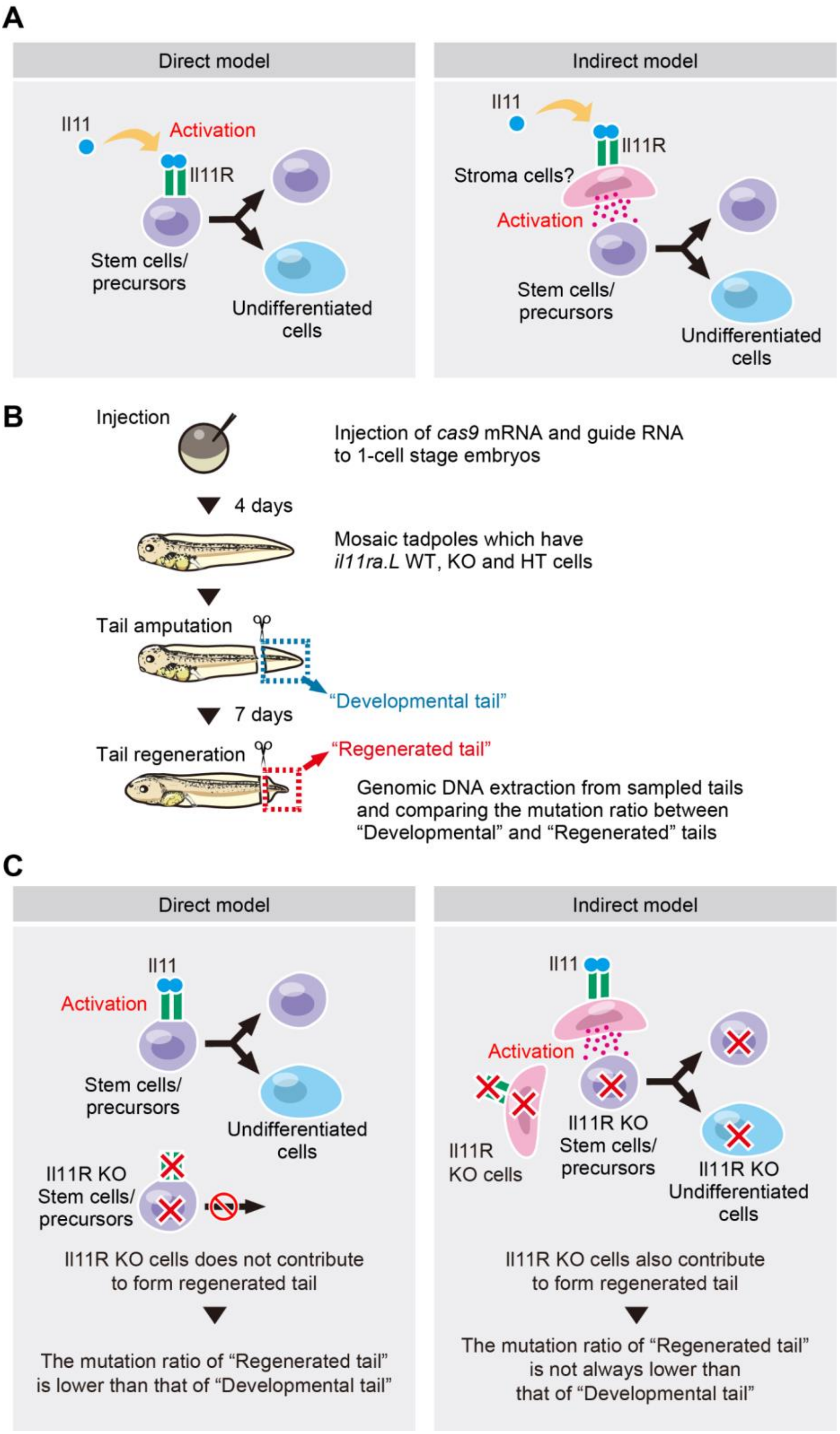

Supplementary Fig. S6
Schematic of the putative mechanism of Il11 function. (A) Putative models of the mechanism of Il11 function. Direct model; Il11 directly activates stem cells or precursors of each tissue in the tail. Indirect model; Il11 triggers downstream events that activate stem cells or precursors of each tissue in the tail. Il11R, Il11 receptor complex. (B) Experimental procedure. (C) Putative behaviors of *il11ra.L* KO cells in both models and results of the mutation ratio comparison.

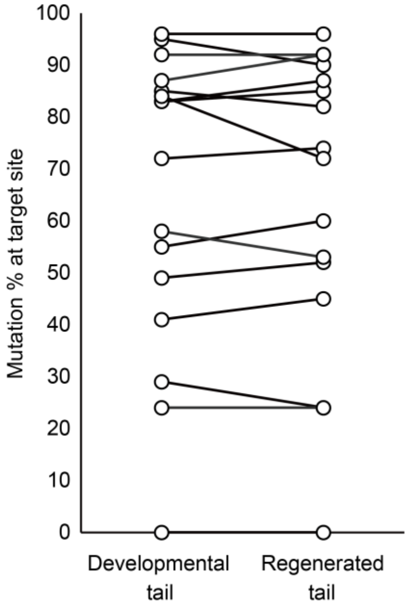

Supplementary Fig. S7

Second result of the mutation ratio estimation of developmental and regenerated tails. n=16. P=0.79, paired t-test.

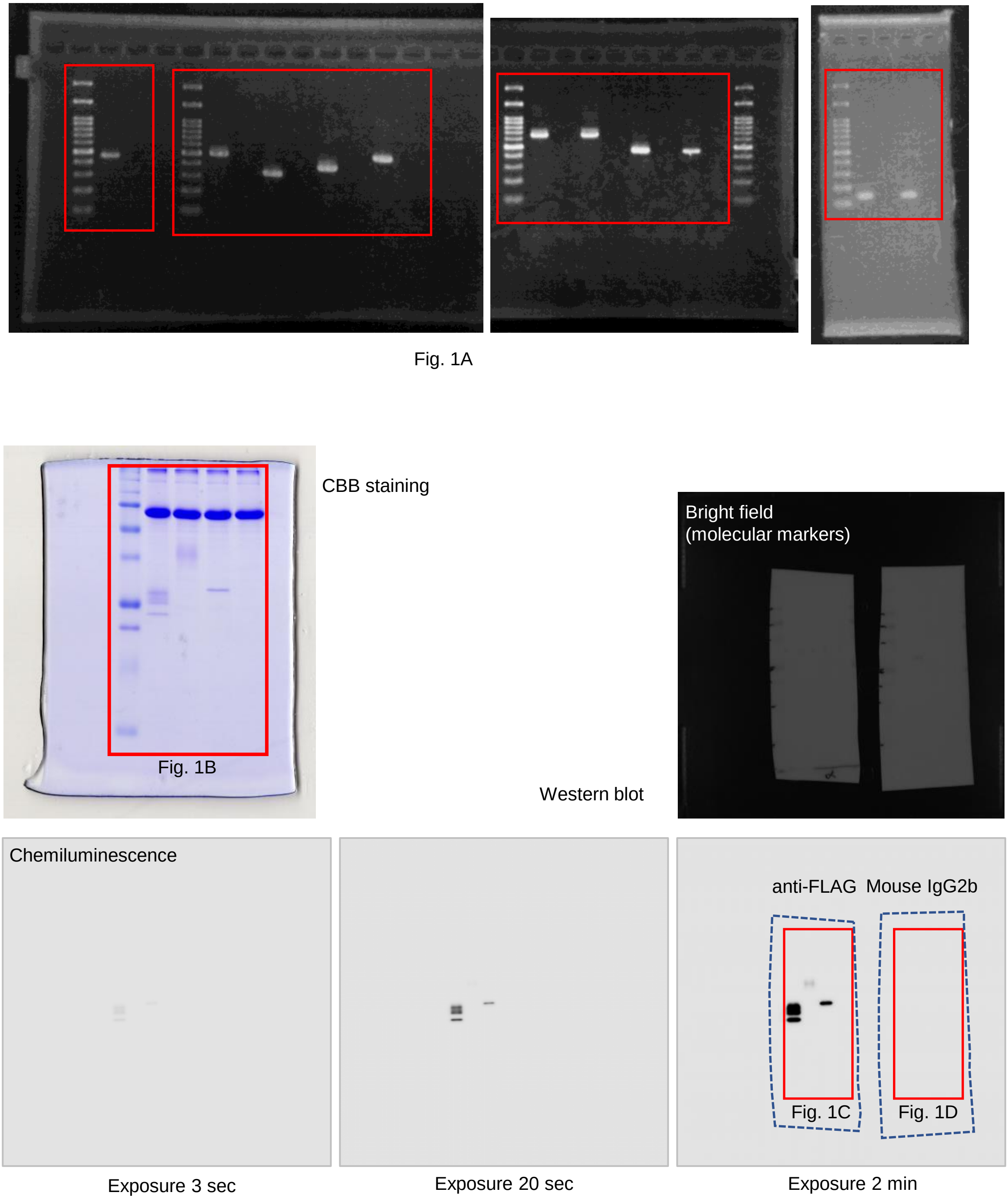

Supplementary Fig. S8
Raw images of full-length gels and blots of Fig. 1.

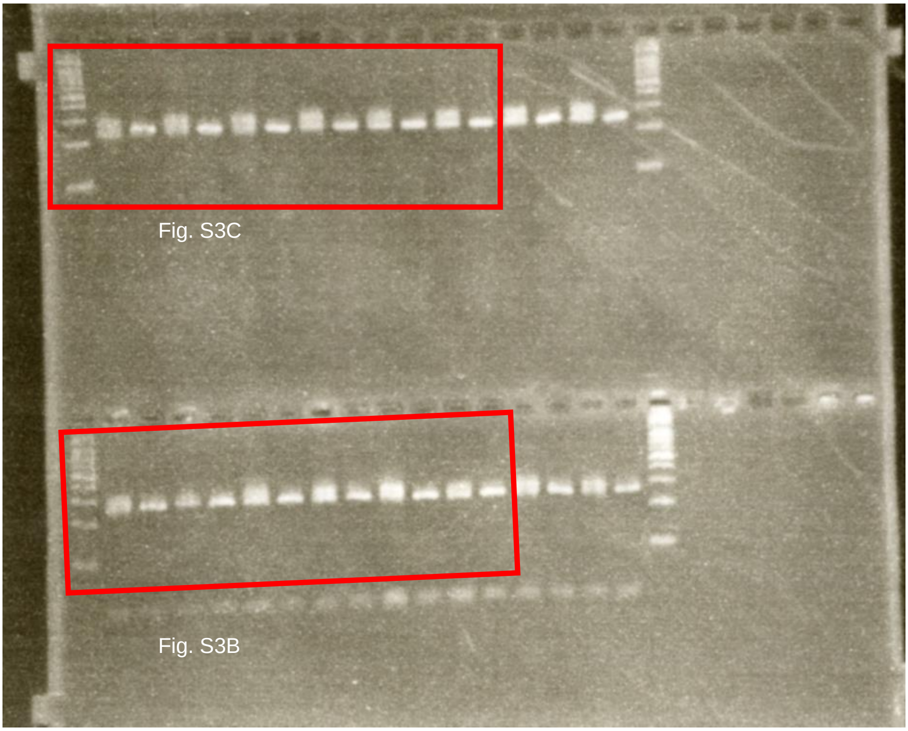

Supplementary Fig. S9

Raw images of full-length gels of Fig. S3.

Supplementary Table S1.

The numbers and ratios of normally developed tadpoles, and tadpoles surviving after tail amputation in *il-11ra.L* KD experiments. Statistical significance was assessed by Fisher's exact test (for Exp.1, 2 and 3, the p values were corrected using the Benjamini-Hochberg method[2]). No significant differences were detected among the 3 (Exp. 1 to 3) or 2 (Exp. 4) groups.

Exp.	groups	Numbers of normally developed tadpoles / injected eggs, and ratios of normally developed tadpoles at 4 dpf	Numbers and ratio of tadpoles surviving after tail amputation
1	*cas9*	68/96 (71%)	68/68 (100%)
	#1 KD	59/96 (62%)	49/55 (89%)
	#1&#2 KD	70/96 (73%)	69/70 (99%)
2	*cas9*	54/96 (56%)	52/54 (96%)
	#1 KD	48/95 (51%)	45/48 (94%)
	#1&#2 KD	49/95 (52%)	49/49 (100%)
3	*cas9*	21/93 (23%)	21/21 (100%)
	#1 KD	23/89 (26%)	22/23 (96%)
	#1&#2 KD	26/88 (30%)	25/26 (96%)
4	*cas9*	25/48 (52%)	25/25 (100%)
	#1&#2 KD	32/48 (67%)	32/32(100%)

2. Benjamini, Y. & Hochberg, Y. Controlling the False Discovery Rate: A Practical and Powerful Approach to Multiple Testing. J. R. Stat. Soc. Ser. B 57, 289–300 (1995).

Supplementary Table S2.

Genes that are significantly upregulated in *cas9* 24 hpa tail stumps but whose upregulation was abolished in tail stumps of the *il11ra.L* KD group, in RNA-seq. FWER, familywise error rate.

Gene name	FWER
rarg-like.1.S	0.0099
wnt5a.L	0.0192
dcbld2.L	0.0307
sept5.L	0.0366
kiaa0020.L	0.0424
fgfrl1.L	0.0487

Supplementary Table S3.

Primer sequences used for RT-PCR of XTC-YF.

genes	primers (5' to 3')	PCR products (bp)
il-11ra.L	AGGAGGCTTCGTGTCAGTT<br>TCCATTCCCTTCTTCCGC	461
il6st.L	CTGTCCAGTGCTCGATCAG<br>GTACAGTGTGTTTCCTTCTAGCTTG	504
il6st.S	ACTGCCAAGTACTCCATCCA<br>CTCCACTTGCTTTGTATGTATGC	335
jak1.L	TGGACCTGAAACTCTCCTCA<br>AATCTTTCAGCGAGTCAAAGC	390
jak1.S	GAGCTGAAGCTTTCCACG<br>GCGACCAACAAGTTCGAG	494
jak2.L	AATACTGCAGGGGAGACTTG<br>TTCAGAGTAGAAAGCTGACTGTAGC	671
jak2.S	TTACCATACTTCAGCAGAGACAGA<br>CAGAGTAGAAAGCCGACTGTAAG	674
stat3.L	GGATTAAACAGTTCTTGCAGAGTA<br>CAAGCTCTTCATCAGTCAGTG	468
stat3.S	TAAACTGTTCCTGCAGAGCC<br>CAAGCTCCTCGTCTGTCAGAT	458
lifr.L	CGTGCAACTGACAAATGATGT<br>CCTCAGCGCAGAGATGTTAG	148
lifr.S	GCAGCTGCCAAATGAAGAT<br>ATTAGCGCTGAGGTGTCCA	147

Supplementary Table S4.

Primer sequences used for quantitative RT-PCR of XTC-YF.

genes	primers (5' to 3')	PCR products (bp)
ef1a	GGAACGGTGACAACATGC<br>AGGCAGACGGAGAGGCTTA	161
il-11ra.L	CCAGATTCACTGCTTCCTACC<br>GAACTCCGACTTCCTGACG	101
il6st.L	GGCTGTGAAAGTGTAAGAAGTGA<br>CTGCAAGATGGCCCTGTA	108
il6st.S	TGGCTGTGAGTGTGTAAGAACTAC<br>ACTGCAAGATGGCCCTATG	109
jak1.L	GAAAGCAAAAGTAAGAACTCTGTTG<br>TCTGGTGAGGAGAGTTTCAGG	160
jak1.S	GAAAGCAAAAGTAAGAACTCAGACA<br>GGCGTGGAAAGCTTCAGC	157
jak2.L	GTCCCGTCCTTGATGACA<br>TGTGATTGGTACAGAAATCCGA	89
jak2.S	CAGAGAGTCCTGTTCTTGATGATG<br>GTGTGATTGGTACTGTTATTCGG	96
stat3.L	CTGAGCTGAATGGCAACAAT<br>CCATGGCTGACAGGAGACTC	129
stat3.S	CTGAGATGAATGGCAACCAA<br>CATGGCTGACAGGAGACTG	128
lifr.L	CGTGCAACTGACAAATGATGT<br>CCTCAGCGCAGAGATGTTAG	148
lifr.S	GCAGCTGCCAAATGAAGAT<br>ATTAGCGCTGAGGTGTCCA	147

For *lifr.L* and *lifr.S*, the same primers were used as for RT-PCR.

Supplementary Table S5.

Primer sequences used for genotyping the *il-11ra.L* KO XTC-YF and heteroduplex mobility assay of KD tadpoles. The same primer sets were used in both experiments.

target	direction	primers (5' to 3')	PCR products (bp)
guide RNA #1	forward	CCTGGTAAACTGCTCTGTGCT	250
	reverse	CTAGACTTGCAAAGGCTGCTTC	
guide RNA #2	forward	CCCTTTGGCACTCAGATCCC	262
	reverse	CCAACGCAGAAGCTAATGATCG	

Supplementary Table S6.

Primer sequences used for estimating mutation ratios of KD embryo tails.

target	direction	primers (5' to 3')	PCR product (bp)
guide RNA #1	forward	TGGTTGTACTCTTACCCAGCA	411
	reverse	TATGCTGCTATAGAGGGTCCGT	

Supplementary Table S7.

Primer sequences used for DIG-labeled probe synthesis.

gene	primers (5' to 3')	PCR product (bp)
il-11ra.L	CCGGGTGATTGTGCTTCTCA<br>TTCTTCCGCACTTTCACCCA	1082
